# Seagrass structural and elemental indicators reveal high nutrient availability within a tropical lagoon in Panama

**DOI:** 10.7717/peerj.11308

**Published:** 2021-05-06

**Authors:** Julie Gaubert-Boussarie, Andrew H. Altieri, J. Emmett Duffy, Justin E. Campbell

**Affiliations:** 1 Institute of Environment, Department of Biological Sciences, Florida International University, Miami, FL, United States of America; 2 Department of Environmental Engineering Sciences, University of Florida, Gainesville, FL, United States of America; 3 Smithsonian Tropical Research Institute, Panama City, Republic of Panama; 4 Tennenbaum Marine Observatories Network, Smithsonian Institution, Edgewater, MD, United States of America

**Keywords:** *Thalassia testudinum*, Nitrogen, Phosphorus, Eutrophication, Bahia Almirante, Bocas Del Toro, Caribbean, Seagrass meadow, Stable isotopes

## Abstract

Seagrass meadows are valued coastal habitats that provide ecological and economic benefits around the world. Despite their importance, many meadows are in decline, driven by a variety of anthropogenic impacts. While these declines have been well documented in some regions, other locations (particularly within the tropics) lack long-term monitoring programs needed to resolve seagrass trends over time. Effective and spatially-expansive monitoring within under-represented regions is critical to provide an accurate perspective on seagrass status and trends. We present a comprehensive dataset on seagrass coverage and composition across 24 sites in Bahía Almirante, a lagoon along the Caribbean coast of Panama. Using a single survey, we focus on capturing spatial variation in seagrass physical and elemental characteristics and provide data on key seagrass bio-indicators, such as leaf morphology (length and width), elemental content (% nitrogen and phosphorus) and stable isotopic signatures (*δ*^13^C and *δ*^15^N). We further explore relationships between these variables and water depth (proxy for light availability) and proximity to shore (proxy for terrestrial inputs). The seagrass assemblage was mostly monospecific (dominated by Thalassia testudinum) and restricted to shallow water (<3 m). Above-ground biomass varied widely, averaging 71.7 g dry mass m^−2^, yet ranging from 24.8 to 139.6 g dry mass m^−2^. Leaf nitrogen content averaged 2.2%, ranging from 1.76 to 2.57%, while phosphorus content averaged 0.19% and ranged from 0.15 to 0.23%. These values were high compared to other published reports for T. testudinum, indicating elevated nutrient availability within the lagoon. Seagrass stable isotopic characteristics varied slightly and were comparable with other published values. Leaf carbon signatures (*δ*^13^C) ranged from −11.74 to −6.70‰ and were positively correlated to shoreline proximity, suggesting a contribution of terrestrial carbon to seagrass biomass. Leaf nitrogen signatures (*δ*^15^N) ranged from −1.75 to 3.15‰ and showed no correlation with shoreline proximity, suggesting that N sources within the bay were not dominated by localized point-source discharge of treated sewage. Correlations between other seagrass bio-indicators and environmental metrics were mixed: seagrass cover declined with depth, while biomass was negatively correlated with N, indicating that light and nutrient availability may jointly regulate seagrass cover and biomass. Our work documents the response of seagrass in Bahía Almirante to light and nutrient availability and highlights the eutrophic status of this bay. Using the broad spatial coverage of our survey as a baseline, we suggest the future implementation of a continuous and spatially expansive seagrass monitoring program within this region to assess the health of these important systems subject to global and local stressors.

## Introduction

Seagrass meadows provide key ecological functions and ecosystem services in coastal zones (Short et al., 2007; United Nations Environment Programme, 2020). These meadows provide shelter and habitat for a variety of marine species, including endangered and iconic species such as sea turtles, manatees and dugongs (Sievers et al., 2019), and their productivity supports coastal food webs (Duarte & Chiscano, 1999). They form ecological connections with other tropical ecosystems such as coral reefs and mangroves (Gillis et al., 2014) and support high-value services such as nutrient cycling, sediment stabilization, and carbon sequestration (Waycott et al., 2009; Fourqurean et al., 2012). Seagrasses are found in shallow coastal waters and estuaries around the world (Short et al., 2007) and although they cover around 0.1–0.2% of the sea floor, they disproportionately provide a large number of services and are among the most valuable ecosystems on the planet (Costanza et al., 1998).

Seagrass meadows are also one of the most threatened habitats (Orth et al., 2006), with global losses estimated at 7% per year towards the end of the 20th century and around 30% of the known areal extent having disappeared since the first records in 1879 (Waycott et al., 2009). This results from local anthropogenic activities such as habitat destruction, overfishing and eutrophication. Other impacts are related to global factors including ocean warming and sea-level rise , which can lead to large-scale trends of seagrasses loss (Duarte, 2002; Waycott et al., 2009). The decline of seagrasses is notable in the Caribbean Sea, where human population expansion and tourism threaten many coastal habitats (Govers et al., 2014; Van Tussenbroek et al., 2014).

In Panama, anthropogenic pressure is particularly noticeable in Bahía Almirante (e.g., Cramer, 2013; Seemann et al., 2018), a coastal semi-enclosed lagoon located on the northwest Caribbean coast of Panama and delimited by the Bocas del Toro archipelago. This region has a long history of banana agriculture and cacao production (Cramer et al., 2012; Fredston-Hermann et al., 2013; Seemann et al., 2014), and is currently experiencing increases in cattle and teak tree farming (Aronson et al., 2014). The resulting deforestation has led to an increase in erosion and sediment runoff, elevating nutrient input into the bay and decreasing water quality (Seemann et al., 2014).

Monitoring coastal habitats is crucial in the context of understanding both the scope and scale of their global decline. Seagrasses can serve as useful indicators of the health of marine habitats and a variety of environmental drivers (Short et al., 2006). In addition to seagrass cover and abundance, the elemental and isotopic composition of seagrass leaf tissue can serve as useful indicators of their nutritional status and surrounding environmental conditions (Fourqurean et al., 2007; Campbell & Fourqurean, 2009). Elemental composition, particularly the content of nitrogen (N) and phosphorus (P), along with calculated N:P ratios, can provide information on the ambient and relative availability of nutrient resources (Fourqurean, Zieman & Powell, 1992). Seagrass leaf stable carbon isotope signatures (*δ*^13^C) can be used as a reliable indicator of (1) benthic light availability and/or (2) inputs of carbon from terrestrial sources (Campbell & Fourqurean, 2009). Further, leaf stable nitrogen isotopic signatures (*δ*^15^N) can be used to infer both the availability and source of dissolved inorganic nitrogen (DIN; Mcclelland, Valiela & Michener, 1997).

Studies of seagrass cover in Bahía Almirante are relatively limited. Through the Caribbean Coastal Marine Productivity Program (CARICOMP, 1998), data on seagrass temporal variability and environmental parameters have been collected since 1998 near the Smithsonian Tropical Research Institute’s station in Bocas del Toro (Guzmán et al., 2005; López-Calderón et al., 2013; Van Tussenbroek et al., 2014). However, CARICOMP was limited to two sites and may not reflect broader seagrass cover across the bay. Another study described *T. testudinum* nutrient content among the three main water bodies of the Bocas archipelago (Carruthers et al., 2005), however biomass and leaf morphometric data were not measured. Thus, bay-wide data on seagrass cover and shoot morphometrics have yet to be published.

In this paper, we present a comprehensive dataset on seagrass percent cover and distribution within Bahía Almirante in Bocas del Toro, Panama. We provide data on the elemental and isotopic signature of surveyed seagrass meadows and compare these data with values reported from other regions in the Caribbean to better understand the health and status of these meadows. These data may be used as baseline measurements for future comparative surveys within this region. Seagrass cover and benthic community composition were surveyed across 24 sites ranging from 0.5 to 8.8 m depth. Aboveground biomass and shoot morphology (shoot area, leaf length and width) of the dominant species, *Thalassia testudinum* are reported, along with leaf tissue elemental (%N, %C, %P, C:N, C:P and N:P ratios) and isotope content (*δ*^13^C and *δ*^15^N). In order to understand some of the mechanisms behind any trends in meadow characteristics, we explored the potential relationships between site water depth (proxy for light availability), site proximity to shore (proxy for potential terrestrial inputs) and our measured variables (e.g., leaf tissue elemental and isotope content, biomass).

## Materials & Methods

### Study sites

**Figure 1:**
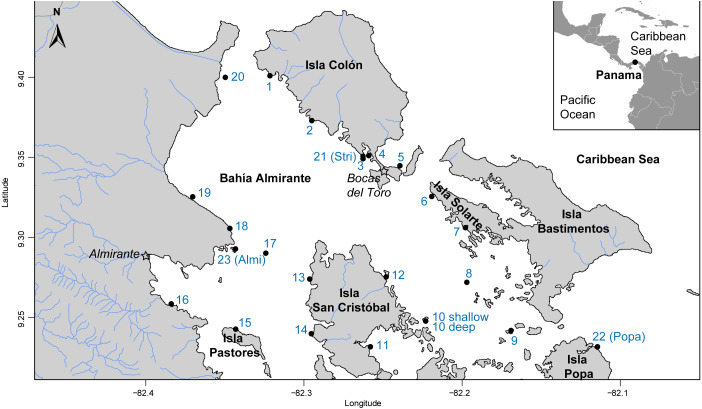
Map of study area. Map of Bahía Almirante in the Bocas del Toro archipelago, Panama showing the 24 sampling sites used in this study (see Table 1 for details).

The Bahía Almirante lagoon is 446 km^2^, extends to a maximum depth of 30 m, and has extensive seagrass meadows, corals reefs and mangroves (Guzmán et al., 2005). Bahía Almirante receives moderate to high freshwater input (mean annual precipitation of approximately 3.3m) and as a result, extensive nutrient loading from the inflowing rivers (Phillips, Rouse & Bustin, 1997; Carruthers et al., 2005). With a minimal tidal range (<0.5 m) and only three narrow connections with the ocean, the water circulation inside the bay is limited (Carruthers et al., 2005; López-Calderón et al., 2013). A total of 24 sites were surveyed across Bahia Almirante (see Fig. 1 and Table S1) in September 2015. Survey sites were selected by superimposing a grid (each cell 1 km^2^) across the entire bay, and GPS coordinates of intersecting grid lines were used to locate 31 potential sites. GPS coordinates with no seagrass coverage were not included in our survey.

### Benthic community composition

At each site, a 25 m transect line was deployed from the site coordinate along a consistent depth, and photographs of replicate quadrats (1 m^2^) were taken every 5m with an underwater camera. Images were later imported into image software (Coral Point Count; Kohler & Gill, 2006), and percent cover of benthic organisms and bare sediment was determined from 50 points randomly superimposed over each photograph. Benthic composition was then identified underneath each point and assigned to one of the following broad taxonomic groups: seagrass, coral, soft coral, sponge, fleshy algae and calcareous algae.

### Seagrass sampling

Seagrass sampling for aboveground biomass (called areal biomass or biomass hereafter) consisted of collecting a single, 15 cm-diameter cylinder core at each site. Seagrass cores were haphazardly located within each meadow and within each core, all aboveground shoot biomass was collected, placed in a labeled bag, and returned to the lab on ice. Seagrasses (predominantly *T. testudinum*) were scraped free of fouling material and the length and width of leaves of all shoots were recorded. Seagrass material was rinsed in deionized water (DI), dried to a constant weight in a 70 °C oven and then dry mass was standardized to a m^2^ basis. An additional 5-6 shoots of the dominant seagrass (*T. testudinum*) were haphazardly harvested along each transect and transported on ice for elemental and isotopic analysis. Afterwards, leaf material was separated, rinsed in DI, scraped free of fouling and dried in a 70 °C oven. Leaf material was ground to a fine powder with a mortar and pestle.

### Elemental and isotopic composition analysis

As seagrasses such as *T. testudinum* are generally long-lived and their tissues integrate nutrient availability across extended time scales, leaf tissue N and P content can serve as a useful gauge of mean ambient nutrient availability relative to spot measurements of water column nutrient concentrations, which can be highly variable (Fourqurean, Zieman & Powell, 1992). Leaf N content was analyzed using a CHN analyzer and leaf P content was determined through dry oxidation and acid hydrolysis extraction followed by a colorimetric analysis (Fourqurean, Zieman & Powell, 1992). Elemental ratios were calculated on a mole:mole basis. A limitation index (LI) was also calculated as follows: LI =|30 − *N*:*P*| (Campbell & Fourqurean, 2009). This index is used to quantify the degree of divergence from the ideal ca. 30:1 ‘Seagrass Redfield Ratio’ (Atkinson & Smith, 1983; Duarte, 1990). Larger LI values indicate greater degrees of nutrient limitation.

Seagrass carbon isotopic composition can also serve as a useful bio-indicator of plant health (overall photosynthetic activity). Prolonged declines in light availability, which reduce photosynthetic carbon demand, result in increased fractionation against the heavier carbon isotope (^13^C) and progressively lighter *δ*^13^C values (Fourqurean et al., 2019). However, it is important to note that shifts in the concentration and source of dissolved inorganic carbon (DIC) can also influence carbon isotope values (Durako & Sackett, 1993; Campbell & Fourqurean, 2011), thus relationships between *δ*^13^C and benthic light availability are strongest when the source and concentration of DIC is relatively constant. For nitrogen, seagrasses fractionate the source pool of DIN dependent upon the relative balance between pool size and plant demand (Fourqurean et al., 2005). Thus, declines in *δ*^15^N may reflect increases in the environmental availability of DIN or reductions in plant demand (potentially related to growth). However, seagrass nitrogen isotope values may also reflect DIN source. Seagrass *δ*^15^N signatures have previously been used as an indicator of wastewater sources of N, as microbial processing increases the *δ*^15^N signature of the source DIN pool and can be reflected in seagrass tissue (Fourqurean et al., 2015).

C and N isotope analyses were conducted using a standard elemental analyzer isotope ratio mass spectrometer procedure. A separate elemental analyzer was used to combust all organic material and subsequently reduce formed gasses into N_2_ and CO_2_, which were measured on a Finnigan MAT Delta C Isotope Ratio Mass Spectrometer in continuous flow mode. The samples’ isotopic ratios (R) are reported in the standard delta notation (*δ*)

\begin{eqnarray*}\delta (\permil )=[({R}_{\mathrm{sample}}/{R}_{\mathrm{standard}})-1]\cdot 1000 \end{eqnarray*}

These results are presented with respect to the international standards of atmospheric nitrogen (N_2_) and Vienna Pee Dee belemnite (V-PDB) for carbon.

We additionally conducted a literature review to compare our leaf N and P content to values reported for *T. testudinum* in other regions across the Caribbean. Relevant literature was searched with the database Google Scholar using the key words (*Thalassia/Thalassia testudinum* and Caribbean), (*Thalassia / Thalassia testudinum* and nutrient or nitrogen or phosphorus).

### Statistical analyses

The normality of benthic community data (% cover per group) and *T. testudinum* elemental and isotopes composition was checked using the Shapiro–Wilk test. Standard linear regressions were used to test the strength of the relationship between (1) depth and % cover or biomass, (2) biomass and %N, %P, (3) shoot area and biomass or depth, and (4) *δ*^13^C or *δ*^15^N and depth or distance to open ocean. Analyses were conducted using tidyverse (Wickham et al., 2019) and ggplot2 (Wickham, 2016) for graphical packages. Non-parametric correlations (Spearman’s *ρ*) were assessed among elemental ratios, limitation index and biomass and water depth. To investigate similarities in the benthic community composition between sites, the benthic community data (% cover per group, after arcsin transformation of these data) was used in a cluster analysis (Bray–Curtis dissimilarity; vegdist and hclust functions). We used Permutational Multivariate Analysis of Variance using distance matrices (PERMANOVA, 999 permutations, vegan package for R (Oksanen et al., 2020) to explore potential factors (e.g., depth, distance from shore) that could explain the benthic community composition. Data visualization and statistics were conducted using R software (version 3.6.2).

## Results

### Benthic community composition across Bahía Almirante

**Figure 2:**
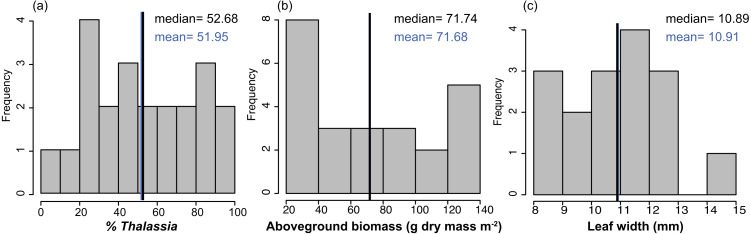
Frequency distributions of seagrass characteristics. Frequency distribution of the % cover, biomass, leaf width of *Thalassia testudinum,* across sites in Bahía Almirante (*n* = 22 for % *Thalassia*, *n* = 24 for the biomass and *n* = 16 for the leaf width). (A) % Thalassia; (B) Aboveground biomass g dry mass m^-2^); (C) Leaf width (mm).

**Figure 3:**
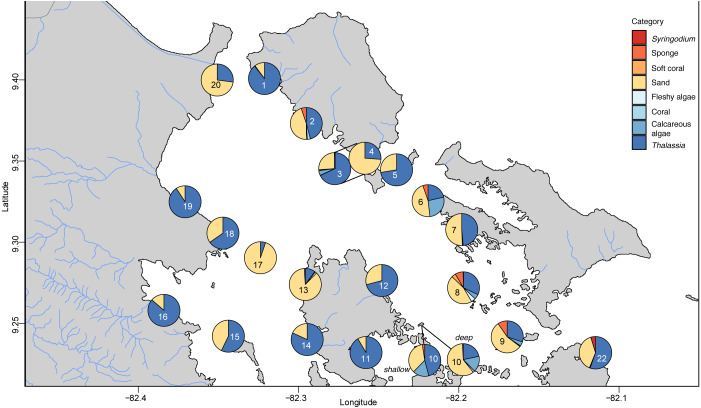
Benthic community composition across the sites throughout Bahía Almirante.

**Figure 4:**
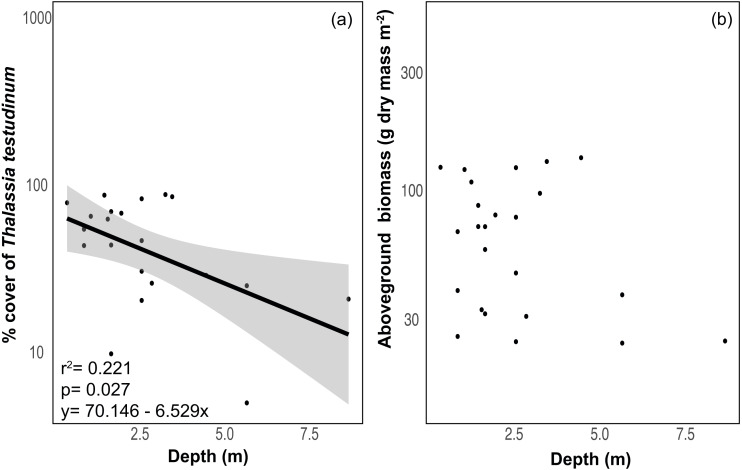
Relationship between depth and seagrass characteristics. Relationship between (A) the % cover of *Thalassia testudinum* (*y* scale is log10 transformed) and depth, and (B) biomass and depth. Linear regression and 95% confidence interval (gray area) of the regression are indicated.

The benthic community in Bahia Almirante was dominated by turtlegrass, *Thalassia testudinum* (mean % cover = 51.95 ± 5.85 SE, Fig. 2), which was present at all seagrass sites. Another seagrass species, *Syringodium filiforme*, was only observed at two sites, with a mean percent cover of 0.24% ± 0.22. Bare space (sediment) was the second major component of benthic cover (mean % cover = 42.02 ± 5.24, Table S2). Other taxonomic groups were observed in smaller abundances and frequency (Fig. 3 , Table S2): calcareous algae (mean % cover = 3.44 ± 1.50), sponges (mean % cover = 1.51 ± 0.61), fleshy algae (mean % cover = 0.46 ± 0.22), soft corals (mean % cover = 0.19 ± 0.19) and stony corals (mean % cover= 0.18 ± 0.14). The % cover of *T. testudinum* was negatively correlated with site depth (*r*^2^ = 0.22, *p* = 0.027, Fig. 4A).

**Figure 5:**
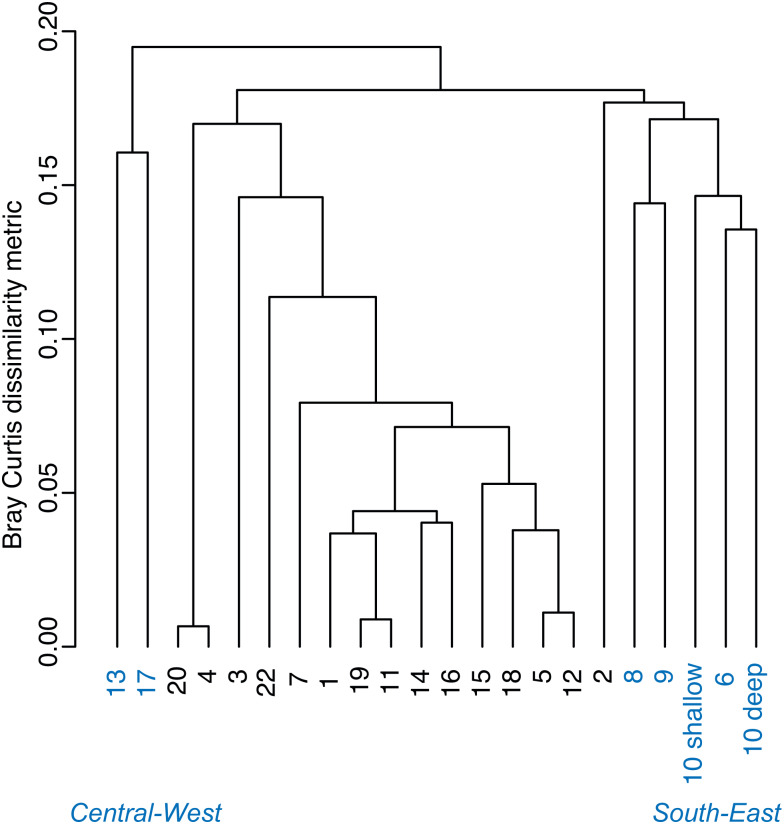
Bray–Curtis dissimilarity cluster analysis of benthic community composition across sites in Bahía Almirante (see Fig. 1).

A Bray–Curtis dissimilarity dendrogram on the benthic community showed three main clusters (Fig. 5). The sites 13 and 17 formed one distinct cluster, as these two sites are in the central-west part of Bahía Almirante where seagrass is sparse and the benthos is primarily composed of bare sediment (86.6 and 94.8% respectively). The second cluster grouped sites from different locations in the bay and across different depths (primarily driven by *Thalassia* dominance). The third cluster on the right of the dendrogram grouped sites from the south-east part of the bay. Compared to the other sites, this third cluster had a more diverse bottom cover, including sponges (percent cover from 0.82 to 9.64%) and calcareous algae (percent cover from 4.42 to 26.64%), along with cover of soft corals (4.08%, one site) and fleshy algae (0.4% to 4.49%) (Fig. 3 and S1). Except for the “central-west” and “south-east” clusters, no clear pattern in the benthic community composition was found among sites according to depth, bay position, distance from the ocean/closest town, the presence of a reserve area or the nutrient content in *T. testudinum* leaf (PERMANOVA, *p* > 0.05; Table S3).

### *Thalassia testudinum* biomass and morphometrics

Mean biomass of *Thalassia testudinum* was 71.7 g dry mass m^−2^ ± 8.1 (± SE), and tended towards a bimodal distribution with values ranging from 24.8 to 139.6 g dry mass m^−2^ (Fig. 2, Table 1). Unlike percent cover, *T. testudinum* biomass was not correlated with site depth (*p* = 0.28, Fig. 4B).

**Table 1:** Seagrass physical characteristics. Percentage cover shoot morphometrics (shoot area, leaf length and leaf width) and biomass (aboveground) of *Thalassia testudinum* across sites in Bahía Almirante (SE, standard error; CV, coefficient of variation).

	% cover of *T. testudinum*	shoot area (mm^2^)	**leaf length** (mm)	**leaf width** (mm)	**Biomass** (g dry mass m^−2^)
Mean	51.95	6,395	190	11	71.7
SE	5.85	970	18	0.4	8.1
CV	0.53	0.61	0.39	0.15	0.56
Median	52.68	6,218	187	11	71.7
Minimum	5.20	656	41	8	24.8
Maximum	91.57	15,690	304	14	139.6
n	22	16	16	16	24

The shoot morphometrics of *T. testudinum* were assessed from a sub-set of sites (16 total). The average shoot area was 6395 ± 970 mm^2^, the average leaf length 190 ± 18 mm and the average leaf width 11 ± 0.4 mm (Table 1). No correlations between shoot morphometrics and areal biomass, depth or nutrient content (*p* > 0.05) were found.

### *Thalassia testudinum* elemental and stable isotope composition

**Figure 6:**
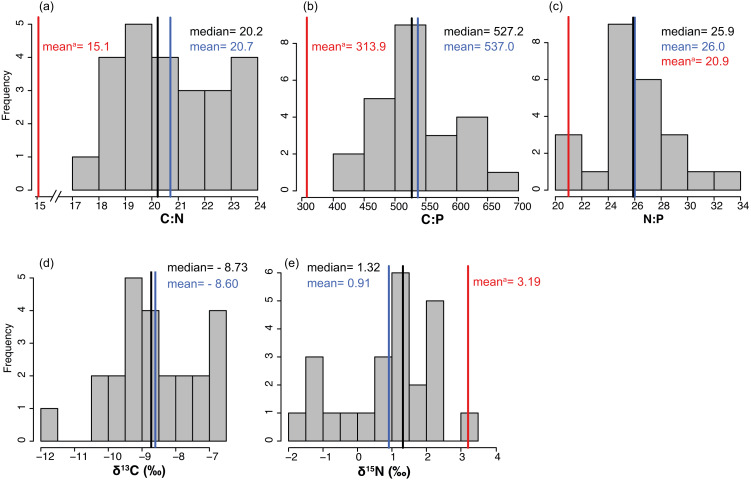
(A-E) Frequency distributions of elemental characteristics. Frequency distributions of elemental (C:N, C:P and N:P) and stable isotope (*δ*^13^C and *δ*^15^N) ratios of *Thalassia testudinum* (*n* = 24). Red lines indicate the mean values of these parameters previously reported in Bahia Almirante (Carruthers et al., 2005).

**Figure 7:**
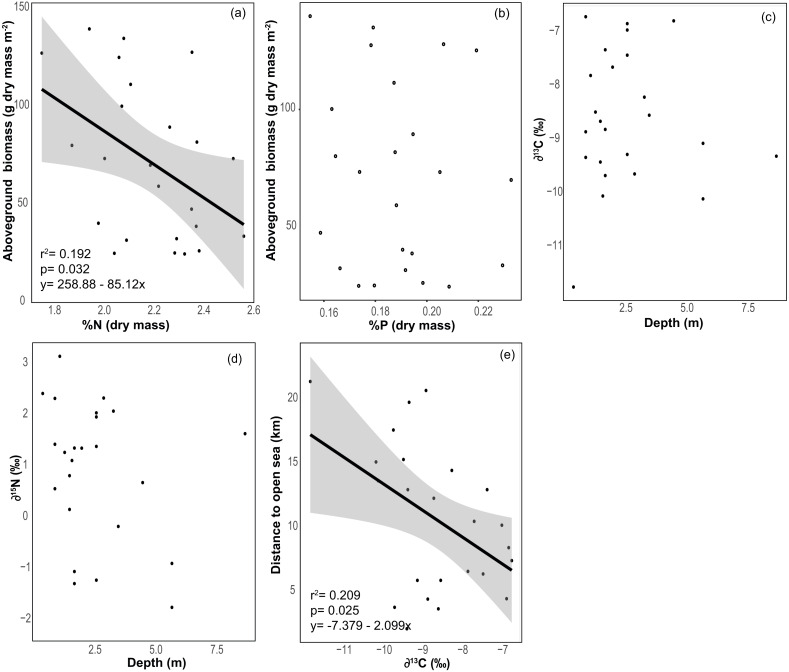
Relationships between seagrass structural and elemental characteristics. Relationship between (A) *Thalassia testudinum* biomass and %N (dry weight), (B) *Thalassia biomass* and %P (dry weight), (C) depth and *δ*^13^C, (D) depth and *δ*^15^N and (E) the distance to the open sea and *δ*^13^C. Linear regression and 95% confidence interval (gray area) of the regression are indicated.

The average % content (dry mass) of C, N and P were 38.77 ± 0.46, 2.20 ± 0.04 and 0.19 ± 0.00 (mean ± SE) respectively, with a low variation among sites (CV = 0.06 to 0.11, Table 2). The frequency distribution of the elemental ratios C:N (mean 20.7 ± 0.39), C:P (mean 537 ± 13.3) and N:P (mean 26.0 ± 0.57, Table 2) displayed a roughly normal distribution (Fig. 6), with a low coefficient of variation among sites (CV = 0.09–0.12). The limitation index (LI) exhibited higher variation among sites (CV =0.52) and had a mean value of 4.30 ± 0.46 (Table 2). Stable carbon isotope values displayed a slight bimodal distribution and varied from −11.74 to −6.7‰ (mean −8.6 ± 0.26‰) and stable nitrogen isotope values from −1.75 to 3.15 ‰ (mean 0.91 ± 0.28‰), and neither were correlated with site depth (Figs. 7C and 7D). The *δ*^13^C was negatively correlated with the distance to the open sea (Fig. 7E) but no similar correlation was found for *δ*^15^N. The %N of *T. testudinum* was negatively correlated with biomass (*r*^2^ = 0.192, *p* = 0.032, Fig. 7A) but no correlation was found between biomass and %P (*p* = 0.57, Fig. 7B). Non-parametric Spearman’s correlations between depth, elemental and stable isotope ratios and LI were explored (Table 3). Of these, depth was only positively correlated with C:P ratio. C:P and C:N were positively correlated, as were C:P and N:P. The *δ*^15^N was positively correlated to C:N and negatively correlated to N:P. No correlation was found between the *δ*^13^C and the elemental ratios.

**Table 2:** Seagrass Elemental Characteristics. Elemental content and stable isotope ratios of *Thalassia testudinum* leaves. (%N, %C and %P are % of dry weight). LI= limitation index, *n* = 24.

	**% N**	**% C**	**% P**	**C:N**	**C:P**	**N:P**	**LI**	*δ*^13^C	*δ*^15^N
Mean	2.20	38.77	0.19	20.7	537.0	26.0	4.30	−8.60	0.91
SE	0.04	0.46	0.00	0.39	13.3	0.57	0.46	0.26	0.28
CV	0.09	0.06	0.11	0.09	0.12	0.11	0.52	0.15	1.49
Median	2.22	39.19	0.19	20.2	527.2	25.9	4.12	−8.73	1.32
Minimum	1.76	34.24	0.15	17.5	441.9	20.9	0.69	−11.74	−1.75
Maximum	2.57	42.18	0.23	23.7	660.6	33.0	9.08	−6.70	3.15

**Table 3:** Correlations among *Thalassia* seagrass characteristics. Correlations (non-parametric Spearman’s *ρ*) among elemental and stable isotope ratios, LI, seagrass biomass and water depth. Correlation coefficients are indicated above the diagonal and the *p*-values (pairwise comparisons) are below. Significant (*p* < 0.05) correlations are indicated in bold. LI: Limitation Index.

	Depth	C:N	C:P	N:P	LI	Biomass	*δ*^13^C	*δ*^15^N
Depth		0.049	**0.461**	0.392	−0.403	−0.167	0.062	−0.266
C:N	0.820		**0.428**	−0.310	0.259	0.266	0.360	0.542
C:P	**0.023**	**0.037**		**0.670**	**−0.646**	−0.041	0.352	−0.109
N:P	0.058	0.141	**<0.001**		**−0.976**	−0.118	0.131	**−0.502**
LI	0.051	0.221	**0.001**	<**0.001**		0.103	−0.095	**0.463**
Biomass	0.436	0.209	0.850	0.582	0.633		0.286	0.190
*δ*^13^C	0.773	0.084	0.091	0.541	0.660	0.175		0.119
*δ*^15^N	0.210	**0.006**	0.613	**0.012**	**0.023**	0.375	0.579	

## Discussion

**Figure 8:**
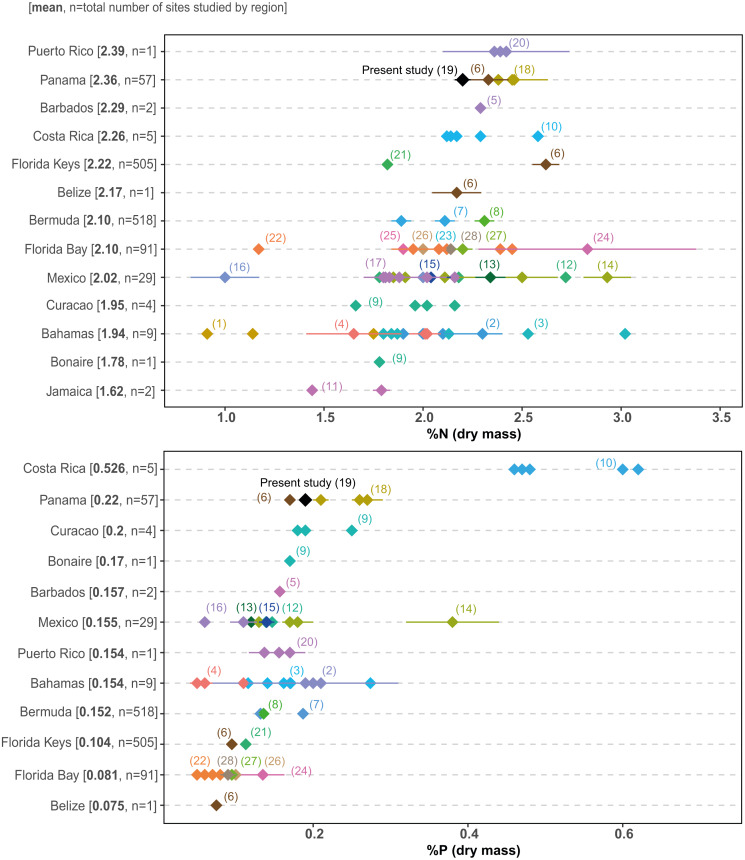
Literature search of *T. testudinum* nutrient content. *Thalassia testudinum* leaf nitrogen (% N) and phosphorus (% P) content from different regions in the Caribbean, taken from the published literature. Regions are ordered by the mean for each nutrient, from the highest to the lowest. Each point represents a studied area for which the mean value (±SE when available) was derived from the indicated reference in parenthesis (see Table S5 for the complete list of references). Numbers in square brackets correspond to the overall mean per region and to the total number of sites studied for this region.

Growing human pressure has led to the decline of many coastal ecosystems in Bahía Almirante (Cramer, 2013; Altieri et al., 2017), where agriculture, deforestation, tourism and urban construction have all triggered declines in the water quality across the bay (Guzmán et al., 2005). Our surveys provide the first comprehensive assessment of seagrass structural (biomass, shoot morphometrics) and chemical characteristics (elemental and isotopic content) and reveal most meadows are (1) dominated by a single species (turtlegrass , *Thalassia testudinum*), (2) restricted to shallow depths (<3m) and (3) display relatively high leaf N and P content. This latter finding is of particular concern, as seagrasses at most sites could be classified as nutrient replete (N:P ratios near 30:1, and leaf N and P content higher than 1.8 and 0.2%, respectively (Atkinson & Smith, 1983; Duarte, 1990). No site displayed the low nutrient content which is commonly observed in tropical seagrasses under oligotrophic settings. Comparisons of seagrass nutrient content across the Caribbean (Fig. 8) supports the relatively eutrophic condition of Bahía Almirante and highlights the vulnerable status of this ecosystem.

The benthic community at all sites was dominated by *T. testudinum* with limited representation from other taxonomic groups (calcareous algae, sponges, fleshy algae, soft corals and hard corals). It has been shown that the seagrass *T. testudinum* is a late successional species which effectively competes for light and nutrients with some rhizophytic algae, a mechanism that potentially explains the low presence of benthic macroalgae in our surveyed meadows (Davis & Fourqurean, 2001). Furthermore, turtlegrass was also most abundant in shallower sites and decreased with depth. Seagrass percent cover rapidly declined to nearly 25% as water depths approached 4 m (Fig. 4A), and most seagrass in the lagoon was restricted to <3 m deep and relatively close to the mangrove shoreline. *Thalassia testudinum* is a large and long-lived species with high below-ground biomass (up to 90% of its total biomass), thus it is restricted to high light environments typically found in relatively shallow depths in order to maintain a positive carbon balance (Fourqurean & Zieman, 1991; Van Tussenbroek et al., 2014). Bahía Almirante is a shallow lagoon, with most of the bay less than 10 m deep, and a maximum depth of 30 m in the ship channel area (Aronson et al., 2014). While we found seagrasses as deep as 8.8 m, coverage and biomass at depth were low. It appears that water-column productivity and rainfall-driven turbidity generally prevent seagrasses from colonizing some of the deeper portions of the bay which receive lower levels of light (Aronson et al., 2014).

Two of three site clusters based on benthic community similarity mapped to areas within the bay. The cluster of two sites located in the central-west portion of the bay were mainly composed of sand (Fig. 3, sites 13 and 17). One of these sites was relatively deep (5.8 m) while the other had heavy epiphytic growth on seagrass. Both factors likely reduced light availability and limited seagrass abundance at this site. The cluster on the southeast part of the bay exhibited higher benthic diversity as compared to most of the other sites, with sponges (1.51 ± 0.61%), calcareous algae (3.44 ± 1.50%) and a small proportion of soft corals and fleshy algae at some sites (0.19 ± 0.19% and 0.46 ± 0.22%, respectively). Due to the distance of the latter sites from the main towns and their proximity to the open sea, it is possible that water quality and recruitment rates may be higher there, resulting in higher benthic diversity. While two sampling sites are in reserve areas (Site 4 is in Matumbal Bay Special Use Zone and Site 8 is in Isla Bastimentos National Marine Park), they did not distinguish from the other sites in terms of benthic community composition or biomass.

The mean bay-wide aboveground biomass of *T. testudinum* (71.7 g dry mass m^−2^) was similar to other reported values at the Panama CARICOMP stations between 1999-2006 (mean: 77.4 g dry mass m^−2^, (Van Tussenbroek et al., 2014) and between 1999–2010 (mean: 66.6 g dry m^−2^; (López-Calderón et al., 2013). Mean *T. testudinum* biomass from our surveys was also similar to values reported at other Caribbean CARICOMP stations (Van Tussenbroek et al., 2014), see Table S4), however, these comparisons should be made with caution as there were generally only 2–4 sites / region in the CARICOMP program, compared to 24 sites in the current study. Thus, while regional means may be informative, our dataset documents large variation in seagrass biomass (24.8–139.6 g dry mass m^−2^) with nearly a third of our sites displaying extremely low values (20–40 g dry mass m^−2^). Our ‘within bay’ variation was similar to the entire reported range of seagrass biomass across all 52 CARICOMP sites (16 –325 g dry mass m^−2^). As CARICOMP is targeted towards documenting long-term trends over time, our survey focused on capturing spatial variation in seagrass physical and elemental characteristics at a single time point, highlighting the distinctions between the two monitoring approaches.

Seagrass biomass displayed a bimodal distribution (Fig. 2), potentially indicating that Bahía Almirante meadows may be broadly classified into one of two categories, either ‘high’ or ‘low’ biomass. Such may be the case if only shallow meadows are able to maintain high biomass, while deteriorating conditions cause deeper meadows to converge towards a lower biomass state because of declining light availability. Such a scenario would be supported by increasingly pronounced bimodal distributions in future surveys. While we documented a significant negative correlation between seagrass % cover and depth, we did not find a strong correlation between biomass and depth. This is because many of our shallow sites displayed high visual percent cover, yet also had low estimates of biomass (Fig. 2), driven by the presence of only a few shoots that were large enough to obscure a substantial portion of the benthic area when viewed overhead. We also observed a negative correlation between seagrass biomass and leaf tissue N content. N enrichment may have increased epiphyte growth and phytoplankton blooms at certain sites, resulting in declining light availability to the leaf surface and consequently decreasing seagrass aboveground biomass (Udy & Dennison, 1997; Lee & Dunton, 2000). Overall, prior work has similarly documented reduced seagrass biomass at depth and under low light conditions (Duarte, 1991), with differences in seagrass ‘high’ and ‘low’ biomass primarily driven by shifts in shoot density, and to a lesser extent, shoot size (Enríquez & Pantoja-Reyes, 2005). Depth limits for *T. testudinum* depend upon overlying water clarity and range from 1.2m –30 m (Duarte, 1991).

Seagrass elemental and isotopic content can give insights into their nutritional status and surrounding environmental conditions (Fourqurean et al., 2007; Campbell & Fourqurean, 2009). Mean elemental ratios for turtlegrass were higher in our study (2015) compared to prior surveys (Carruthers et al., 2005): C:N = 20.60 vs 15.1, C:P = 537.0 vs 313.9 and N:P = 26.0 vs 20.9 (see S3 for a spatial comparison between survey sites). These slight increases may indicate a broader trend of reduced nutrient availability across the bay over the past decade. However, we note that as compared to other regions in the Caribbean, many of the meadows in Bahía Almirante are still extremely nutrient replete (see Fig. 8 for a literature overview). The N and P content of *T. testudinum* found in our study was near the high range of values reported from other areas (e.g., Bahamas, Belize, South Florida, Caribbean coast of Mexico). It has been hypothesized that nutrient availability in Bahia Almirante is partly the result of high rainfall and runoff from a highly impacted watershed, which has been subjected to both agriculture and deforestation (Carruthers et al., 2005; Guzmán et al., 2005). In our study, no spatial pattern of seagrass nutrient content was observed, thus we did not find any nutrient gradient from the mainland to the open ocean, similar to other findings (Carruthers et al., 2005), suggesting that nutrient availability across the bay is relatively homogeneous. The Bahía Almirante is relatively small, with a low tidal range (<0.5 m) and only three narrow connections with the open ocean (Carruthers et al., 2005; López-Calderón et al., 2013), thus bay flushing is limited relative to the delivery of nutrients from the inflowing creeks.

The mean N:P ratio (26 ± 0.57) was near the proposed ‘Redfield’ ratio for seagrasses (30:1; Atkinson & Smith, 1983; Duarte, 1990), suggesting that seagrass growth was likely not strongly limited by either N or P. The measured N:P ratio may be explained by the late successional status of this species, its slow growth rate, high ambient nutrient availability and large below-ground biomass which can facilitate the access to sediment nutrient pools (Williams, 1987; Campbell & Fourqurean, 2009). Given the nutrient-replete status of all sites, it is likely that the deeper meadows, which are characterized by low seagrass cover, are light-limited.

Depth was positively correlated to C:P ratio, however this relationship was weak (coefficient = 0.46). The *δ*^15^N of *T. testudinum* was not correlated to site depth, however we note that this was being driven by the presence of one outlier site (Fig. 3F). When re-analyzed without this site, we found a significant negative relationship between depth and leaf *δ*^15^N, which is expected as deeper meadows with lower productivity can discriminate more strongly against the heavier nitrogen isotope. This is supported by the significant positive correlation between leaf *δ*^15^N and C:N ratios. Overall, most leaf *δ*^15^N values were relatively light (<2 ‰), likely driven by a combination of both high N availability and potentially a light DIN pool from agricultural sources (such as the banana farms in the Bocas watershed), as artificial fertilizer displays a *δ*^15^N near 0‰ (Chalk, Inácio & Magalhães, 2014).

Not a single site could be characterized as nutrient-limited within our survey. Median LI (4.12) was similar to values previously reported for this species in the Florida Keys (6.2), but our maximum value was five times lower than the maximum value reported in the Florida Keys (9.08 vs 46.5), suggesting high nutrient availability throughout the entire bay. Seagrasses are generally dominant in oligotrophic conditions and as nutrient availability increases, other primary producers with higher nutrient demands (macroalgae, epiphytic microalgae, phytoplankton) can benefit and potentially out-compete seagrasses for light (Fourqurean & Rutten, 2003). As *T. testudinum* in Bahía Almirante appeared nutrient replete at all studied sites, a further enrichment of the bay may be detrimental via competition with faster growing species that could tip the system towards an algal-dominated state.

The mean *δ*^13^C of *T. testudinum* leaves is within the range reported for this seagrass (Lin, Banks & da Silveria Lobo O’Reilly Sternberg, 1991), and contrary to what was expected (see methods), no correlation was found between depth and *δ*^13^C or *δ*^15^N. We only document a correlation between *δ*^13^C and the distance to the open sea, with an increase of *δ*^13^C in the sites closer to the open sea. Bahía Almirante is a high rainfall area, surrounded by extensive mangrove forests (Guzmán et al., 2005), and this proximity to mangroves influences the inorganic carbon sources into the bay, potentially influencing the *δ*^13^C of seagrass leaf tissue (Lin, Banks & da Silveria Lobo O’Reilly Sternberg, 1991). Respiratory CO_2_ from the decomposition and mineralization of mangroves detritus has low *δ*^13^C values. Therefore, the relationship between depth and *δ*^13^C may have been obscured by the influence of a relatively light carbon source. Moreover, in the open sea, *δ*^13^C of the DIC pool tends to be close to 0‰ (Lloyd, 1964; Hoefs, 1987; Lin, Banks & da Silveria Lobo O’Reilly Sternberg, 1991), thus sites in closer proximity to the open sea may have been exposed to a heavier DIC pool and exhibited higher (less negative) leaf *δ*^13^C as compared to sites closer to shore.

## Conclusions

The elemental composition (high N and P content) and depth-restricted distribution of seagrasses in Bahia Almirante suggest that these waters are relatively eutrophic. Comparisons with other published values across the Caribbean reveal that seagrasses in the Bocas Del Toro region rank as second highest regarding N and P content. Combined with the finding that most seagrass cover is limited to <3 m depth, it is possible that many meadows may be approaching, or have already surpassed, a ‘tipping point’ towards seagrass loss, in which the first indicators would be deeper meadows losing all coverage and/or shallow meadows becoming sparsely populated with high epiphytic growth. Using our surveys as a baseline dataset, we encourage the establishment of a comprehensive and spatially-expansive monitoring program to assess the future health and trajectory of these important systems. While the Bocas Del Toro watershed has historically been highly impacted, we remain optimistic that these meadows have the potential to recover, dependent upon future efforts to mitigate precipitation-driven nutrient input from the surrounding watershed. Given that many meadows globally have experienced declines, multi-faceted monitoring programs are needed to understand why some have displayed surprising resilience (O’Leary et al., 2017) serving as ecological “bright spots” in the face of environmental disturbance.

##  Supplemental Information

10.7717/peerj.11308/supp-1 Supplemental Information 1 Supplemental materials

10.7717/peerj.11308/supp-2 Supplemental Information 2 Raw seagrass survey data

## Additional Information and Declarations

### Data Availability

The following information was supplied regarding data availability: The raw survey data is available in the Supplemental File.
