# Supplementary material for: Seagrass structural and elemental indicators reveal high nutrient availability within a tropical lagoon in Panama
Source: PeerJ. 2021 May 6;9:e11308. doi: 10.7717/peerj.11308 (PMC8106914; doi:10.7717/peerj.11308)
Supplement: Supplemental Information 1 [file peerj-09-11308-s001.pdf]

SUPPLEMENTARY INFORMATION

Seagrass structural and elemental indicators reveal high nutrient availability within a tropical lagoon in Panama.

Julie Gaubert-Boussarie[1], Andrew H. Altieri[2,3], J. Emmett Duffy[4], Justin E. Campbell[1*]

[1]Department of Biological Sciences, Florida International University, Marine Sciences Program, 3000 NE 151 Street, North Miami, Florida 33181, USA.

[2] Department of Environmental Engineering Sciences, University of Florida, Gainesville, Florida, 32611, USA

[3] Smithsonian Tropical Research Institute, Apartado Postal 0843-03092, Panamá, Republic of Panamá

[4]Tennenbaum Marine Observatories network, Smithsonian Institution, Edgewater, MD 21037, USA

**Corresponding author*

Table S1. Sampling sites across the Bahía Almirante, used to survey the benthic community cover and the nutrient content of *Thalassia testudinum*.

Site ID	Depth (m)	Latitude	Longitude
1	3.6	9° 24.049'N	82° 19.275'W
2	1	9° 22.388'N	82° 17.709'W
3	1.2	9° 20.951'N	82° 15.722'W
4	5.8	9° 21.104'N	82° 15.513'W
5	1.8	9° 20.674'N	82° 14.331'W
6	2.7	9° 19.534'N	82° 13.150'W
7	2.7	9° 18.466'N	82° 11.890'W
8	2.7	9° 16.314'N	82° 11.824'W
9	4.6	9° 14.500'N	82° 10.188'W
10 shallow	1.8	9° 14.918'N	82° 13.386'W
10 deep	8.8	9° 14.918'N	82° 13.386'W
11	3.4	9° 13.919'N	82° 15.474'W
12	2.1	9° 16.581'N	82° 14.880'W
13	1.8	9° 16.437'N	82° 17.747'W
14	0.5	9° 14.401'N	82° 17.676'W
15	1	9° 14.526'N	82° 20.626'W
16	2.7	9° 15.480'N	82° 23.038'W
17	5.8	9° 17.424'N	82° 19.426'W
18	1.7	9° 18.335'N	82° 20.829'W
19	1.6	9° 19.493'N	82° 22.247'W
20	3	9° 23.995'N	82° 21.044'W
21 (Stri)	1.4	9°21.144'N	82°15.500W
22 (Popa)	1	9°13.913'N	82°06.883W
23 (Almi)	1.6	9° 17.453'N	82° 20.580'W

Table S2. Benthic community composition (% cover) across sites in Bahia Almirante (n=22).

	% *Thalassia*	% *Syringodium*	% Sand	% Coral	% Soft coral	% Sponge	% Fleshy algae	% Calcareous algae
Mean	51.95	0.24	42.2	0.18	0.19	1.51	0.46	3.44
SE	5.85	0.22	5.24	0.14	0.19	0.61	0.22	1.50
CV	0.53	4.32	0.58	3.73	4.69	1.90	2.25	2.05
Median	52.68	0.00	41.48	0.00	0.00	0.00	0.00	0.00
Minimum	5.20	0.00	8.43	0.00	0.00	0.00	0.00	0.00
Maximum	91.57	4.77	94.80	3.09	4.08	9.64	4.49	26.64

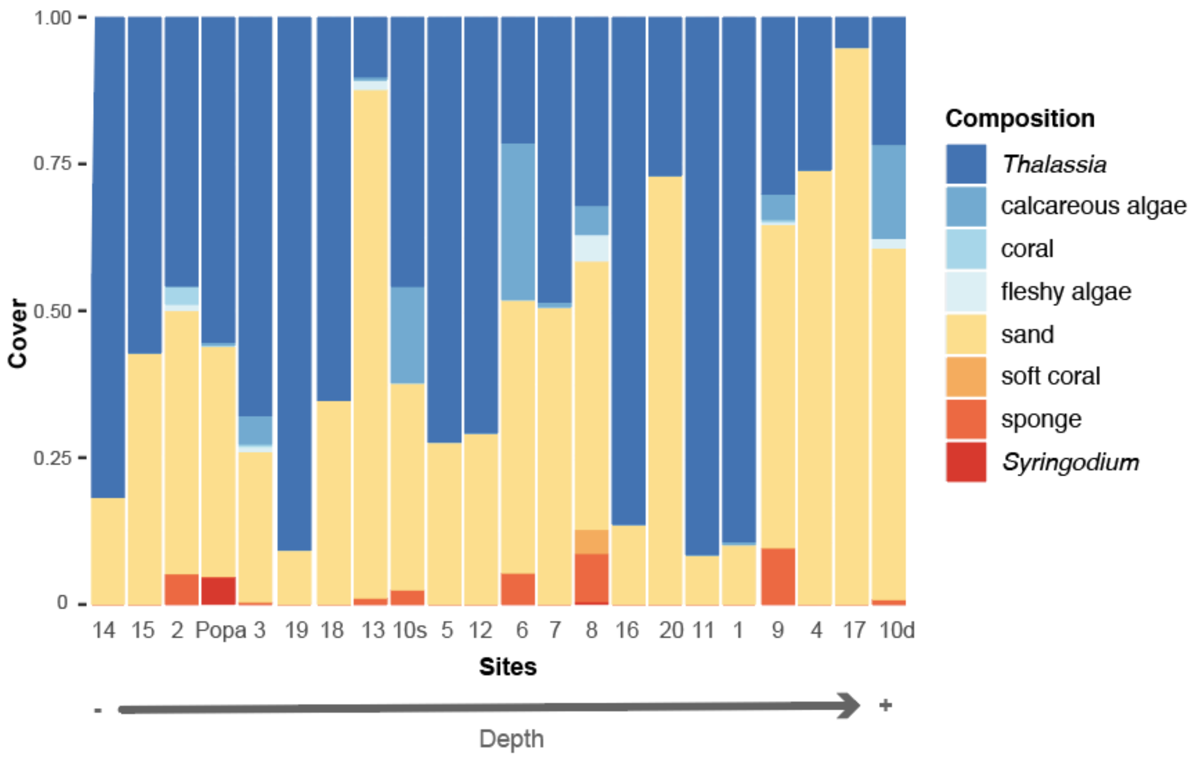

Figure S1. Visual benthic community cover according to sites (from the shallower to the deepest sites).

Table S3. Results of Permanova tests (999 permutations) exploring the effect of different factors on the benthic community cover according to sites.

Response variable	F	Pr(>F)
Latitude	0.67	0.420
Longitude	1.16	0.293
Distance from open ocean	0.55	0.488
Distance from the closest city	0.54	0.510
Depth	3.74	0.070
Management	0.17	0.765
Leaf %N	0.27	0.641
Leaf %C	0.69	0.421
Leaf %P	0.12	0.857

Figure S2. (A) Elemental ratios, biomass and limitation index (LI) values and (B) stable isotope ratios (δ^{13}C and δ^{15}N) values across the Bocas del Toro archipelago.

(A)

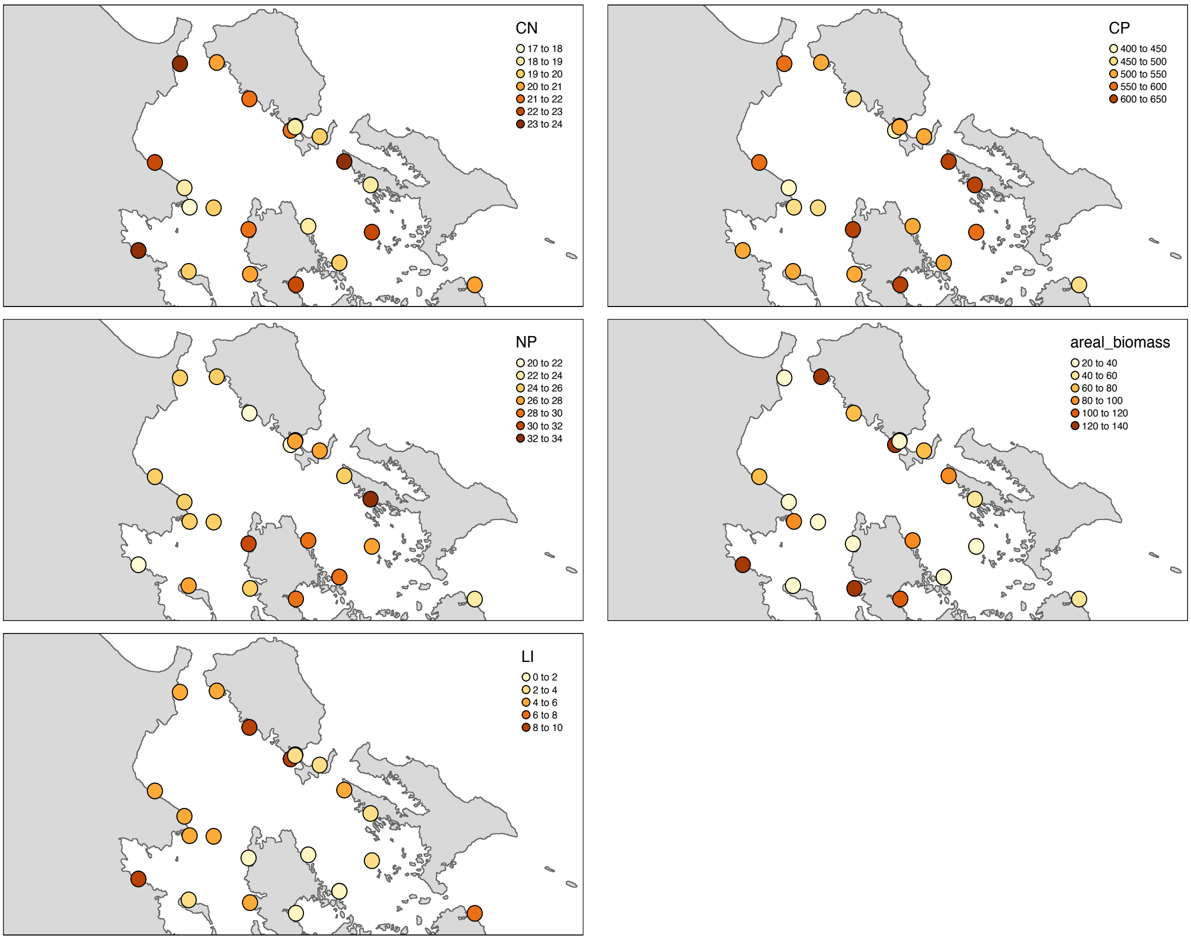

(B)

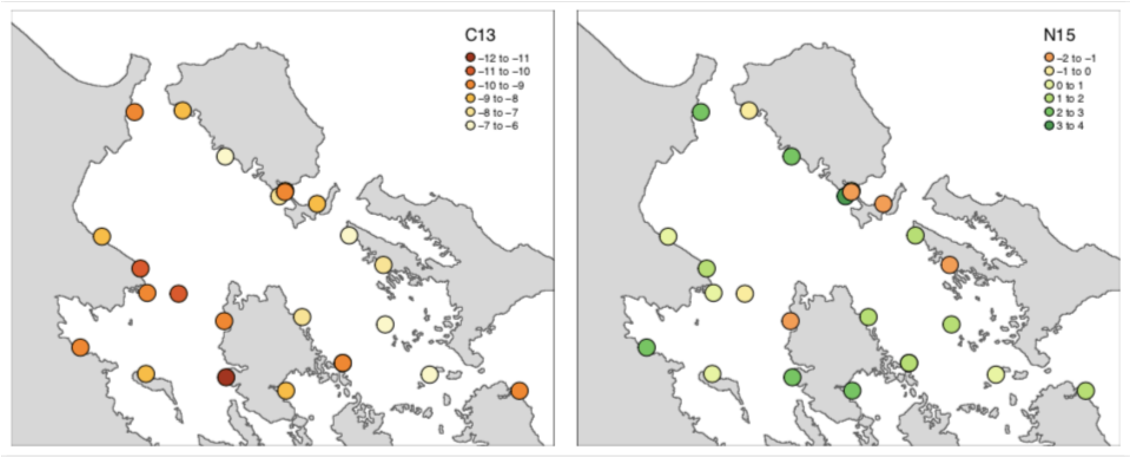

Table S4. Average aboveground biomass of *Thalassia testudinum* at the CARICOMP seagrass monitoring sites per country or territory, summarized from van Tussenbroek *et al.* 2014. The last line (in grey) reports the average aboveground biomass found in Bahía Almirante in this study.

Country/Territory (*number of stations)*	Period of study	Average above-ground biomass of *T. testudinum* (g dry mass m^{-2})
**Bermuda** *(3)*	1993-2000	9.3
**Bahamas** *(2)*	1994-2006	51.1
**Cuba** *(2)*	1994-2002	73.0
**Mexico** *(5)*	1993-2009	71.5
**Cayman Isl.** *(2)*	1997-2003	68.8
**Jamaica** *(2)*	1993-1999	120.7
**Dominican R.** *(2)*	1996-2001	63.1
**Puerto Rico** *(2)*	1994-2007	131.9
**Belize** *(4)*	1993-2012	89.4
**Colombia (Isla Providencia and Isla San Andres)** *(10)*	1999-2007	61.3
**Colombia** *(2)*	1994-2005	66.8
**Barbados** *(2)*	1993-2001	76.8
**Curaçao** *(2)*	1994-1995	22.9
**Tobago** *(3)*	1992-2007	77.4
**Venezuela** *(3)*	1993-2006	154.9
**Costa Rica** *(2)*	1999-2005	69.1
**Panama** *(2)*	1999- 2006	77.4
**Panama (Bahia Almirante,** *24***)**	2015	71.68

Table S5. References associated with Figure 8, reviewing *T. testudinum* mean leaf %N and %P values according to different regions in the Caribbean. The number of sites associated with each mean value reported is indicated.

Country	Ref	Authors	Number of sites
Bahamas	1	Capone *et al.* 1979	2
Bahamas	2	Moran & Bjorndal 2007	1
Bahamas	3	Jensen *et al.* 1998	3
Bahamas	4	Allgeier *et al.* 2011	3
Barbados	5	Patriquin 1972	2
Belize	6	Campbell *et al.* 2017	1
Bermuda	7	Mcglathery *et al.* 1994	2
Bermuda	8	Fourqurean *et al.* 2015	516
Bonaire	9	Govers *et al.* 2014a	1
Costa Rica	10	Krupp *et al.* 2009	5
Curacao	9	Govers *et al.* 2014a	4
Jamaica	11	Peterson *et al.* 2012	2
Mexico (Caribbean coast)	12	Gallegos *et al.* 1993	3
Mexico (Caribbean coast)	13	Hernández & Tussenbroek 2014	2
Mexico (Caribbean coast)	14	Carruthers *et al.* 2005a	18
Mexico (Caribbean coast)	15	Van Tussenbroek *et al.* 1996	1
Mexico (Caribbean coast)	16	Mutchler *et al.* 2007	2
Mexico (Caribbean coast)	17	Pérez-gómez *et al.* 2020	3
Panama	18	Carruthers *et al.* 2005b	18
Panama	18	Carruthers *et al.* 2005b	9
Panama	18	Carruthers *et al.* 2005b	5
Panama	6	Campbell *et al.* 2017	1
Panama	19	Present study	24
Puerto Rico	20	Olsen & Valiela 2010	1
Florida Keys	21	Fourqurean & Zieman 2002	504
Florida Keys	6	Campbell *et al.* 2017	1
Florida Bay	22	Armitage *et al.* 2011	6
Florida Bay	23	Kenworthy & Thayer 1984	1
Florida Bay	24	Powell *et al.* 1989	1
Florida Bay	25	Rublee & Roman 1982	1
Florida Bay	26	Fourqurean & Cai 2001	31
Florida Bay	27	Fourqurean *et al.* 1992a	50
Florida Bay	28	Campbell *et al.* 2012	1

Figure S3. Map of Bahía Almirante showing the seagrass sites used in this study (in black) and in the study of Carruthers *et al.* (2005 ; in red).

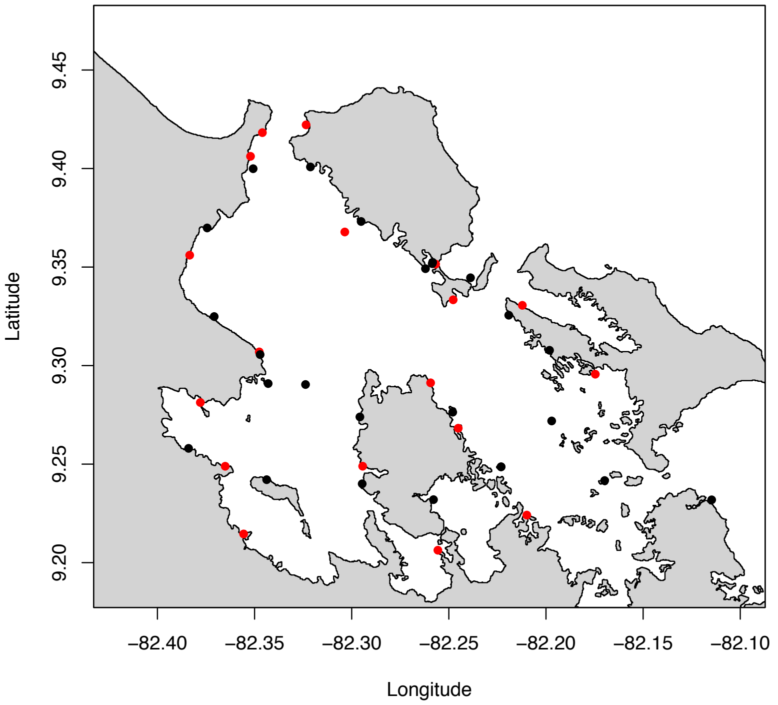

References

Allgeier, J.E., Rosemond, A.D. & Layman, C.A. (2011). Variation in nutrient limitation and seagrass nutrient content in Bahamian tidal creek ecosystems. *J. Exp. Mar. Bio. Ecol.*, 407, 330–336.

Armitage, A.R., Frankovich, T.A. & Fourqurean, J.W. (2011). Long-Term Effects of Adding Nutrients to an Oligotrophic Coastal Environment. *Ecosystems*, 14, 430–444.

Campbell, J.E., Altieri, A.H., Johnston, L.N., Kuempel, C.D., Paperno, R., Paul, V.J., Duffy, J.E. (2017). Herbivore community determines the magnitude and mechanism of nutrient effects on subtropical and tropical seagrasses. *J. Ecol.*, 00, 1–12.

Campbell, J.E., Yarbro, L.A. & Fourqurean, J.W. (2012). Negative relationships between the nutrient and carbohydrate content of the seagrass *Thalassia testudinum*. *Aquat. Bot.*, 99, 56–60.

Capone, G., Penhale, P.A. & Taylor, F. (1979). Relationship between productivity and N_2 (C_2H_2) fixation in a *Thalassia testudinum* community. *Limnol. Oceanogr.*, 24, 117–125.

Carruthers, T.J.B., Tussenbroek, B.I. Van & Dennison, W.C. (2005a). Influence of submarine springs and wastewater on nutrient dynamics of Caribbean seagrass meadows. *Estuar. Coast. Shelf Sci.*, 64, 191–199.

Carruthers, T.J.B.C., Barnes, P.A.G.B., Jacome, G.E.J. & Fourqurean, J.W.F. (2005b). Lagoon Scale Processes in a Coastally Influenced Caribbean System : Implications for the Seagrass *Thalassia testudinum*. *Caribb. J. Sci.*, 41, 441–455.

Fourqurean, J.W. & Cai, Y. (2001). Arsenic and phosphorus in seagrass leaves from the Gulf of Mexico. *Aquat. Bot.*, 71, 247–258.

Fourqurean, J.W., Manuel, S.A., Coates, K.A., Kenworthy, W.J. & Boyer, J.N. (2015). Water quality, isoscapes and stoichioscapes of seagrasses indicate general P limitation and unique N cycling in shallow water benthos of Bermuda. *Biogeosciences*, 12, 6235–6249.

Fourqurean, J.W. & Zieman, J.C. (2002). Nutrient content of the seagrass *Thalassia testudinum* reveals regional patterns of relative availability of nitrogen and phosphorus in the Florida Keys USA. *Biochemistry*, 61, 229–245.

Fourqurean, J.W., Zieman, J.C. & Powell, G.V.N. (1992a). Phosphorus limitation of primary production in Florida Bay : Evidence from C : N : P ratios of the dominant seagrass *Thalassia testudinum*. *Limnol. Oceanogr.*, 37, 162–171.

Gallegos, M.E., Merino, M., Marba, N. & Duarte, C.M. (1993). Biomass and dynamics of *Thalassia testudinum* in the Mexican Caribbean : elucidating rhizome growth. *Mar. Ecol.*, 95, 185–192.

Govers, L.L., Lamers, L.P.M., Bouma, T.J., Brouwer, J.H.F. De & Katwijk, M.M.V. (2014a). Eutrophication threatens Caribbean seagrasses – An example from Curaçao and Bonaire. *Mar. Pollut. Bull.*, 89, 481–486.

Hernández, A.L.M. & Tussenbroek, B.I. Van. (2014). Patch dynamics and species shifts in seagrass communities under moderate and high grazing pressure by green sea turtles. *Mar. Ecol. Prog. Ser.*, 517, 143–157.

Jensen, H.S., Mcglathery, K.J., Hall, C., Marino, R. & Howarth, R.W. (1998). Forms and availability of sediment phosphorus in carbonate sand of Bermuda seagrass beds. *Limnol. Oceanogr.*, 43, 799–810.

Kenworthy, W.J. & Thayer, G.W. (1984). Production and decomposition of the roots and rhizomes of seagrasses, *Zostera marina* and *Thalassia testudinum*, in temperate and subtropical marine ecosystems. *Bull. Mar. Sci.*, 35, 364–379.

Krupp, L.S., Cortés, J. & Wolff, M. (2009). Growth dynamics and state of the seagrass *Thalassia testudinum* in the Gandoca-Manzanillo National Wildlife Refuge, Caribbean, Costa Rica. *Rev. Biol. Trop.*, 57, 187–201.

Mcglathery, K.J., Marino, R., Howarth, R.W., Mcglathery, K.J. & Howarth, W. (1994). Ecological Significance Variable rates of phosphate uptake by shallow marine carbonate sediments : Mechanisms and ecological significance. *Biogeochemistry*, 25, 127–146.

Moran, K.L. & Bjorndal, K.A. (2007). Simulated green turtle grazing affects nutrient composition of the seagrass *Thalassia testudinum*. *Mar. Biol.*, 150, 1083–1092.

Mutchler, T., Dunton, K.H., Townsend-small, A., Fredriksen, S. & Rasser, M.K. (2007).

Isotopic and elemental indicators of nutrient sources and status of coastal habitats in the Caribbean Sea, Yucatan Peninsula, Mexico. *Estuar. Coast. Shelf Sci.*, 74, 449–457.

Olsen, Y.S. & Valiela, I. (2010). Effect of Sediment Nutrient Enrichment and Grazing on Turtle Grass *Thalassia testudinum* in Jobos Bay, Puerto Rico. *Estuaries and Coasts*, 33, 769–783.

Patriquin, D.G. (1972). The origin of nitrogen and phosphorus for growth of the marine angiosperm *Thalassia testudinum. Mar. Biol.*, 15, 35–46.

Pérez-Gómez, J.A., García-Mendoza, E., Olivos-Ortiz, A. & Paytan, A. (2020). Indicators of nutrient enrichment in coastal ecosystems of the northern Mexican Caribbean. *Ecol. Indic.*, 118, 106756.

Peterson, B.J., Stubler, A.D., Wall, C.C. & Gobler, C.J. (2012). Nitrogen-rich groundwater intrusion affects productivity, but not herbivory, of the tropical seagrass *Thalassia testudinum. Aquat. Biol.*, 15, 1–9.

Powell, G.V.N., Kenworthy, W.J. & Fourqurean, J.W. (1989). Experimental evidence for nutrient limitation of seagrass growth in a tropical estuary with restricted circulation. *Bull. Mar. Sci.*, 44, 324–340.

Rublee, P.A. & Roman, M.R. (1982). Decomposition of turtlegrass (*Thalassia testudinum* König) in flowing sea-water tanks and litterbags: compositional changes and comparison with natural particulate matter. *J. Exp. Mar. Bio. Ecol.*, 58, 47–58.

van Tussenbroek, B.I., Hermus, K., Tahey, T., (1996). *Thalassia testudinum* (Banks ex König) biomass and growth in a shallow tropical lagoon system, in relation to tourist development. *Caribbean Journal of Science*, 32, 357e364.
